# Method for quantitative and qualitative evaluation of hazardous waste in laboratories of Isfahan University of Medical Sciences, Iran

**DOI:** 10.1016/j.mex.2019.02.012

**Published:** 2019-02-16

**Authors:** Hamidreza Pourzamani, Mohammad Darvishmotevalli, Sepideh Habib Akhyari, Susan Hadi, Faezeh Momeni, Setareh Gashul Bakhtiyari, Saeid Fadaei

**Affiliations:** a Environment Research Center, Research Institute for Primordial Prevention of Non-communicable disease, Isfahan, Iran; b Student Research Committee, Faculty of Health, Isfahan University of Medical Sciences, Isfahan, Iran

**Keywords:** Determination of quantitative and qualitative of hazardous waste in laboratories, Waste management, Hazardous waste, Chemical and biological laboratories, Iran

## Abstract

Hazardous wastes (HWs) is waste that has substantial or potential threats to public health or the environment. The aim this study is evaluation of quantitative and qualitative of hazardous waste in laboratories of Isfahan University of Medical Sciences (IUMS), Isfahan, Iran. In this data research, four out of ten faculties of IUMS that produce the highest amount of HWs were selected for collection of required data. The required information collected by using interview with laboratory staffs, completion of questionnaire, and refer to the available documents. The 33 laboratories (including 18 and 15 chemical and biological laboratories, respectively) and one clinic were selected. The obtained data showed that annually 2609.9 kg HWs generated in IUMS. The dentistry, public health, medical, and pharmacy faculties were produced 104, 266.6, 793.9, and 1445.4 kg of HW per year, respectively that they were including 4%, 10%, 31%, and 55% in the total amount of generated HW. According to the acquired result, to protect the health of the environment, IUMS must establish a comprehensive solid waste management programs for prevention and reduction, higher rates of recycling, and managing HWs in all faculties.

## Graphical abstract

**Figure:**
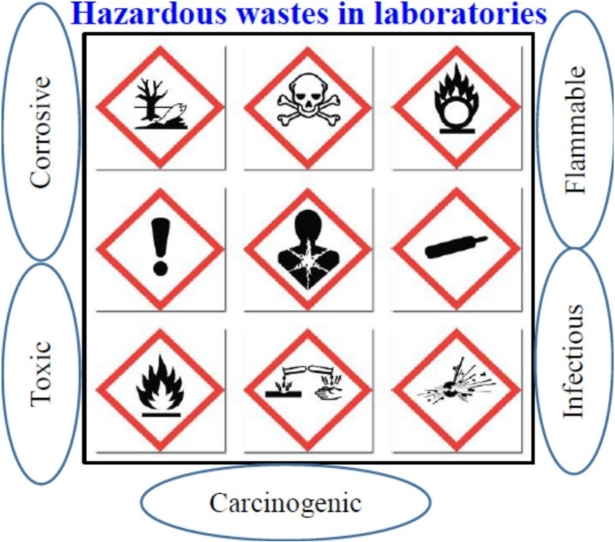

Specifications table

**Table:** 

Subject area	Environmental Health Engineering
More specific subject area	Hazardous waste management,
Methods name	The applied method in this study is determination of quantitative and qualitative of hazardous waste in medical laboratories. The required information collected by using interview with laboratory staffs, completion of questionnaire, and refer to the available documents. The 33 laboratories (including 18 and 15 chemical and biological laboratories, respectively) and 1 clinic were selected for performance of this data research.
Name and reference of original method	M.S. Hassanvand, K.Naddafi, R.Nabizadeh, F.Momeniha, A. Mesdaghinia, K.Yaghmaeian, Hazardous waste management in educational and research centers: a case study. Toxicol. Environ.Chem. 93 (2011) 1636-42(Published) [1].
Resource availability	The data are available with this article.

## Method details

### Sampling area

**Fig. 1:**
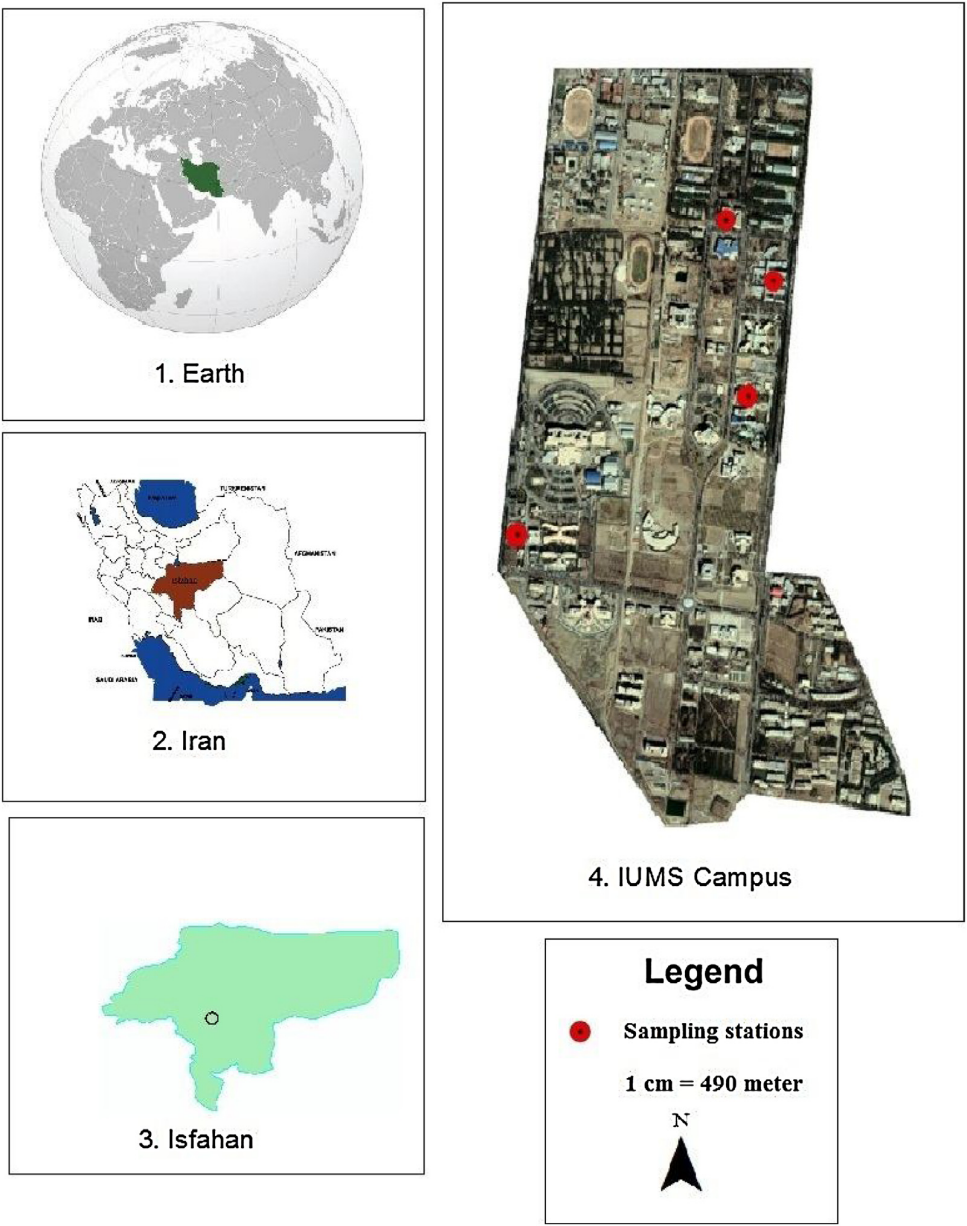
The geographical location of IUMS.

Isfahan University of Medical Sciences (IUMS) is a university specializing in basic medical sciences, clinical sciences, and health services that located in Isfahan, Iran. IUMS has located in same place by an area of 100 ha and has 10 faculties and 8720 students, approximately. Major activities on campus focused on teaching, research, and community services. Fig. 1 shows the location of IUMS.

### Data collection

At first, in this data research, four out of ten faculties of IUMS that produce the highest amount of hazardous wastes (HWs) were selected for collection of required data. The required information collected by using interview with laboratory staffs, completion of questionnaire, and refer to the available documents. The 33 laboratories (including 18 and 15 chemical and biological laboratories, respectively) and 1 clinic were selected. To determine the total amount of HWs at IUMS, liquids, semisolid, and solid HWs were considered; finally, assuming that 1 L of HWs is almost equivalent to 1 kg, the total amount of HWs was expressed in mass unit.

We used a questionnaire that designed by Hassanvand et al. [1] that is consist of five sections: (1) Quantity of HWs generation, (2) Separation, packaging, and labeling of HWs, (3) temporary storage method of HWs, (4) Discharge or collection frequency of HWs, and (5) treatment and final disposal method of HWs [[2], [3], [4], [5], [6], [7], [8]].

### Hazardous solid classification

In this data research, hazardous solid wastes classified according to United States of America (USA) Environmental Protection Agency that defined in below [[5], [6], [7], [8], [9], [10], [11], [12], [13], [14]].The HWs that classified in several groups, we select one of character and has been shown in Table 1.

• **Ignitability:** Ignitable wastes can create fires under certain conditions. Examples include liquids that readily catch fire, substances which are friction-sensitive or cause fire through absorption of moisture and ignitable compressed gases.
• **Corrosively:** Corrosive wastes include those that are strongly acidic or basic and those that are capable of corroding metal (such as containers, drums and barrels).
• **Reactivity**: Reactive wastes are unstable under normal conditions. They can create explosions, toxic fumes, gases, and vapors when mixed with water or heated in confinement.
• **Toxicity**: Toxic wastes are harmful or fatal when ingested or absorbed. The toxicity can be chronic or acute. Toxic wastes can cause carcinogenic, mutagenic and teratogenicity effects on human or other life forms.

**Table 1:** The classification o HWs at this data research.

Flammable	Carcinogenic	Infectious	Toxic	Corrosive
• Ethanol • Acetone • Hexane • Butanol • Ethyl acetate	• Methanol • Chloroform • Chlorinated Compounds • Ethidium Bromide	• Solid and liquid growth medium • Sharps, syringes, needles • Feces and urine • Animals corpse • Single-use latex gloves • Petri dishes • Tissue	• Ethylenediaminetetraacetic acid • Acrylamide • Reagents (e.g. phosphate, nitrate and etc.) • Heavy metals	• Ammoniac • All acids (e.g. Nitric, Acetic and etc.)

## Result

**Fig. 2:**
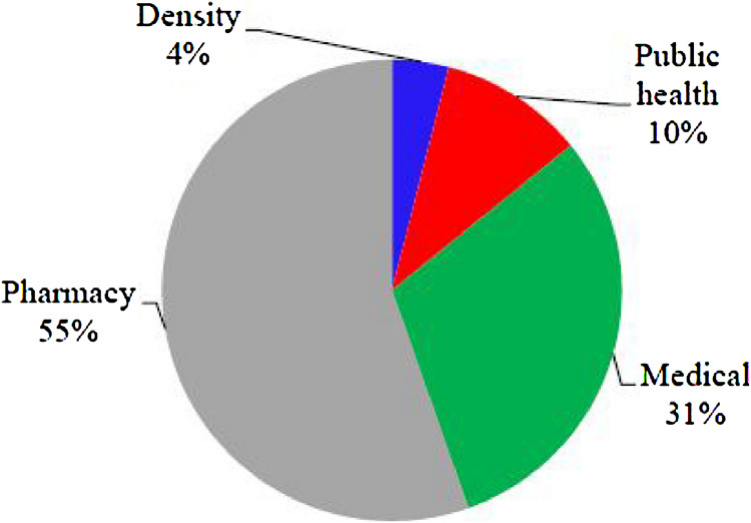
The portion of each faculty in HWs production in IUMS.

**Fig. 3:**
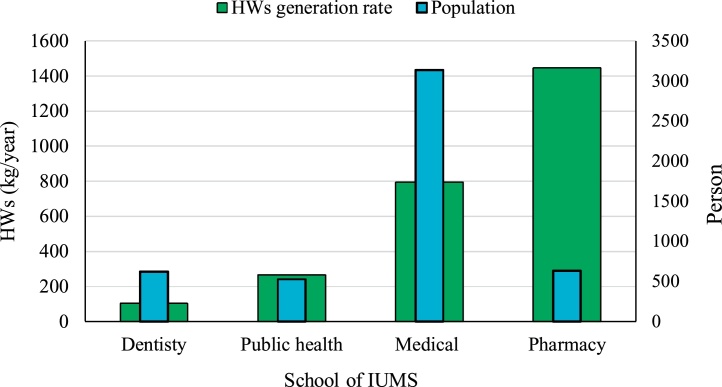
The HWs production rate and population of the faculties.

**Fig. 4:**
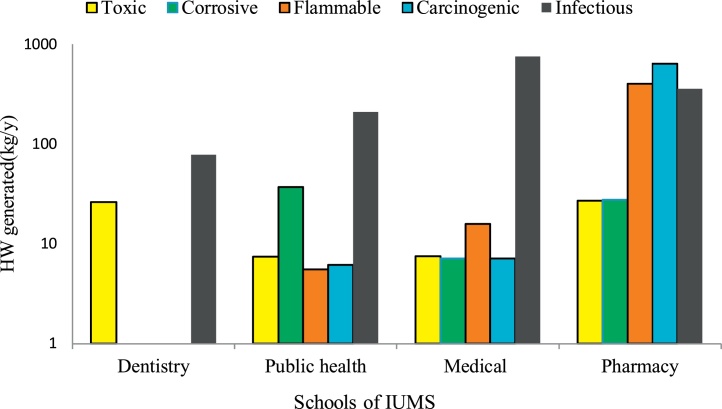
The type and amount of HWs generated in faculties.

The characteristics of the investigated clinic and laboratories was showed in Table 2. Table 3 illustrates the generation rate of hazardous wastes (HWs) at the Isfahan University of Medical Sciences (IUMS). It shows that annually 2609.9 kg HWs generating in IUMS except for HWs that discharges to wastewater. The dentistry, public health, medical, and pharmacy faculties produced 104, 266.6, 793.9, and 1445.4 kg HWs per year, respectively that they are include 4%, 10%, 31%, and 55% in the total amount of generated HWs at IUMS (Fig. 2). Fig. 3 shows the HWs production rate and population of the investigated faculties of IUMS. The dentistry, public health, medical, and pharmacy faculties produce 0.17, 0.5, 0.25, and 2.29 kg HWs/Capital. Year, respectively. Based on provided data in Fig. 4, in all faculties (except pharmacy), the highest share of HWs is related to the infectious type, while about pharmacy faculty, the amount of carcinogenic type is highest, although its difference was insignificant with infectious type. In term of HWs management, in all of the faculties, HWs including liquid or solid types, culture media, bloody waste, and needles is stored in containers temporarily. Usually, all HWs is collected weekly, but according to the type of HWs and its generation rate, sometimes daily and monthly. Some of the generated waste like culture media has the highest amount in faculties that autoclaved in situ and was collected with household wastes. But other HWs after autoclaving, is transferred to landfilling location by truck. Unfortunately, liquid HWs discharges into the municipal wastewater system and only the few laboratories is stored this waste type and have a specific plan.

**Table 2:** The characteristics of the investigated clinic and laboratories.

Characteristics and variables		Frequency
Number of laboratory and clinic	Chemical laboratory	18
	Microbial laboratory	14
	clinic	1
Education degree of laboratory head	Bachelor (B.S)	4
	Master of sciences (MSc.)	22
	Ph.D	7
Daily work hours	<4	0
	4–6	4
	>6	29
Time period of activity	Permanent	29
	Temporary	1
	Seasonal	3
Laboratory area (m^2^)	<100	21
	100–200	9
	>200	3

**Table 3:** Generation rate of HWs in IUMS.

Schools	Produced HWs (kg/year) in different characteristics					
	Toxic	Corrosive	Flammable	Carcinogenic	Infectious	Total
Dentistry	26.0	–	–	–	78.0	104.0
Public health	7.4	36.9	5.5	6.1	210.6	266.6
Medical	7.5	7.1	15.8	7.1	756.5	793.9
Pharmacy	27.0	27.5	399.8	633.2	357.8	1445.4
Total	67.9	71.5	421. 1	646.4	1402.8	2609.9
